# Cell Phone Counseling Improves Retention of Mothers With HIV Infection in Care and Infant HIV Testing in Kisumu, Kenya: A Randomized Controlled Study

**DOI:** 10.9745/GHSP-D-18-00241

**Published:** 2019-06-24

**Authors:** Avina Sarna, Lopamudra Ray Saraswati, Jerry Okal, James Matheka, Danmark Owuor, Roopal J. Singh, Nancy Reynolds, Sam Kalibala

**Affiliations:** a Population Council, New Delhi, India.; b Population Council, New Delhi, India. Now with Research Triangle Institute, New Delhi, India.; c Population Council, Nairobi, Kenya.; d Population Council, Kisumu, Kenya.; e Population Council, Delhi, India.; f Johns Hopkins School of Nursing, Baltimore, MD, USA.; g Population Council, Washington, DC, USA.

## Abstract

**Background:**

We evaluated the effectiveness of a cell phone counseling intervention to promote retention in care and HIV testing of infants among women with HIV accessing prevention of mother-to-child services in Kisumu, Kenya.

**Methods:**

Between May 2013 and September 2015, we recruited 404 pregnant women with HIV who were between 14 and 36 weeks of gestation and randomly assigned them to the intervention (n=207) or control arm (n=197). Retention was assessed at delivery and at 6 and 14 weeks postpartum. We also measured uptake of infant HIV testing. The intervention comprised a fixed protocol of counselor-delivered phone calls to provide one-to-one need-based support. The number of calls made varied depending on when participants presented for antenatal care services; the maximum number was 42. The control group received routine care. We evaluated retention at 3 time points using the complementary log-log regression model taking into account factors associated with retention and loss to follow-up time. We calculated the incidence rate for HIV transmission among infants and used binary logistic regression to identify predictors of HIV infection among infants.

**Results:**

Participants attended on average 63% of the required number of counseling calls during the study period. Retention was higher in the intervention arm than the control arm at delivery (95.2% vs. 77.7%, respectively); 6 weeks postpartum (93.9% vs. 72.9%, respectively); and 14 weeks postpartum (83.3% vs. 66.5%, respectively) (*P*<.001). The counseling intervention (hazard ratio [HR]=0.29; 95% confidence interval [CI]=0.12, 0.69) and positive health perceptions (HR=0.99; 95% CI=0.98, 1.00) were associated with lower hazards of being lost to follow-up. HIV testing of infants was higher in the intervention than control arm (93% vs. 68%, respectively; *P*<.001). In total, 9 of 308 (2.9%) infants tested positive for HIV infection (incidence rate=0.39 infections/100 infant-weeks). Medication Possession Ratio (MPR) >90%, used to assess adherence to ART, was associated with lower odds of a positive HIV test among infants (adjusted odds ratio=0.20; 95% CI=0.04, 0.99). Attendance at antenatal and postnatal care visits was higher among participants in the intervention arm than the control arm.

**Conclusions:**

The one-on-one tailored counseling delivered via cell phone was effective in retaining mothers with HIV infection in care and promoting uptake of infant HIV testing and antenatal and postnatal care services. Phone counseling offers a practical approach to reach and retain pregnant women with HIV infection and postpartum mothers in care, but greater emphasis on collection of medications and adherence is required.

## Abstract (precis)

Tailored, one-on-one counseling delivered via cell phone was very effective in retaining mothers with HIV in care and in promoting infant HIV testing and antenatal and postnatal care attendance. The highest risk of loss to follow-up among women with HIV accessing PMTCT services was prior to delivery and then after infant HIV testing at 6 weeks. Challenges include continued limited access to cell phones, difficulty with reaching participants on the phone, and poor adherence to antiretroviral therapy for a substantial percentage of the population.

## INTRODUCTION

The *Global Plan Towards the Elimination of New HIV Infections Among Children by 2015 and Keeping Their Mothers Alive* (Global Plan) was launched in 2011. The 2015 Global Plan progress report for Kenya revealed that 70% of pregnant women living with HIV received antiretroviral therapy (ART).^1^ There were 13,000 new infections among children in 2014, with a mother-to-child transmission rate of 5% at 8 weeks and 17% when including breastfeeding. In addition, the 2013 Kenya AIDS Indicator Survey reports an HIV prevalence of 6.1% among women who had a birth in the 5 years preceding the survey.^2^ Mother-to-child transmission remains a challenge, with poor retention rates for mothers in care and consequently less-than-complete uptake of testing for early infant diagnosis (EID) between 6 and 8 weeks of age.^3^

A series of recent systematic reviews^4^^–^^6^ report a variety of interventions implemented in lower-middle-income countries to promote retention of mother-baby pairs and uptake of HIV testing and ART among exposed infants. Vrazo et al. (2018)^6^ found interventions focused on antenatal care (ANC) and ART integration, family-centered approaches, and the use of lay health care providers to be demonstrably effective in increasing service uptake and retention in care. Geldsetzer et al. (2016)^5^ report that overall the evidence base for interventions to improve postpartum retention in HIV care is weak, except for some evidence that phone-based interventions have a positive impact during the first 3 months postpartum.^5^ Evaluations of interventions such as male involvement,^7^ mother-to-mother peer groups/mentor mother programs,^8^ community health worker support,^9^ and continuous quality improvement^10^ have provided varying results. While the mentor mother intervention was successful in improving mothers' retention in care (retention was 61.9% in the mentor mother arm compared with 24.9% in the control arm), the male involvement, community health worker support, and continuous quality improvement interventions did not show any effect. Other quality improvement projects using rapid results initiative^11^ and health system redesign,^12^ as well as other community health worker interventions^13^^,^^14^ using individualized community-based follow-up, were successful in improving ART initiation among mothers with HIV and their infants, but did not specifically examine retention rates of mothers in care, especially during the antenatal period and beyond 6 weeks postpartum.

With the widespread availability of mobile phones there is increased interest in the use of technology-based methods to improve health services uptake for better health outcomes among mothers with HIV and their exposed children. Ambia et al.'s systematic review of 34 studies^4^ includes 5 studies (2 of which are randomized) that evaluated mobile phone-based interventions that showed a significant increase in the uptake of EID at 6 weeks.^3^^,^^15^^–^^18^ Three studies conducted in Kenya have evaluated mobile phone-based interventions to improve retention, adherence to treatment, and uptake of HIV testing for infants; however, almost all of these interventions have used short message service (SMS) as reminders^3^^,^^15^^,^^16^ that show an improvement in the uptake of infant HIV testing but not in the retention of mothers in HIV care. Counseling support delivered via cell phones has been shown to improve adherence among people living with HIV who are on ART.^19^^,^^20^ One-on-one counseling interventions based on self-regulation have been evaluated for adherence to ART among people living with HIV in the United States.^19^^–^^21^ Similar counseling interventions have also been used for smoking cessation among people living with HIV in the United States.^22^ There are, however, no studies that have used mobile phones to deliver one-on-one counseling support for pregnant women living with HIV and who are accessing prevention of mother-to-child transmission (PMTCT) services.

There is increased interest in using technology-based methods to improve health services uptake among mothers with HIV and their exposed children.

We evaluated the effectiveness of a structured cell phone counseling intervention, informed by behavioral theory and delivered by trained counselors, on maternal retention in care until 14 weeks after birth and uptake of EID/HIV testing in Kisumu, Kenya. The project was called the Healthy Mother Healthy Baby Project.

We evaluated the effectiveness of structured cell phone counseling on maternal retention in care and uptake of infant HIV testing in Kisumu, Kenya.

## METHODS

### Study Design and Study Sites

We conducted a parallel-group, unblinded, randomized controlled study among pregnant women living with HIV who were accessing PMTCT services in Kisumu, Kenya. The primary objective of our study was to determine whether a structured, counselor-delivered, tailored cell phone counseling intervention would increase (1) retention in care until 14 weeks postpartum, and (2) uptake of EID or infant HIV polymerase chain reaction (PCR) testing. As secondary outcomes, we examined HIV transmission among HIV-exposed infants and maternal attendance at ANC and postnatal care (PNC) services.

The study was conducted at 14 HIV treatment clinics providing PMTCT services in Kisumu County. High-volume sites were selected in consultation with County AIDS Control officials from a list of clinics providing PMTCT services under the AIDS, Population and Health-Integrated Assistance (APHIA) Plus Program in Kisumu, which is supported by the United States Agency for International Development. All clinics provided similar PMTCT services per the national protocol with regard to provision of antiretroviral (ARV) medications and client follow-up. Between May 2013 and September 2015, pregnant women living with HIV were recruited and randomly assigned to the intervention and control arms using computer-generated random numbers. Participants were followed up to 14 weeks postpartum.

### Eligibility Criteria

We invited pregnant women living with HIV who were between 14 and 36 weeks of gestation, aged ≥16 years, residing in Kisumu and planning to stay there for the next 12 months, willing and able to provide consent, and who had access to a cell phone (owned or shared) to participate in the study. Participants could be ART naïve or experienced (they were currently on ART or had received nevirapine for a previous pregnancy). Clinic nurses informed potential participants about the study, obtained verbal consent, and then introduced them to the study staff for completing consent and recruitment procedures.

### Study Visits

All study visits were linked to routine maternal and child health services: monthly ANC visits before delivery (the national program recommends a minimum of 4 scheduled comprehensive ANC visits during pregnancy), PNC visit at 6 weeks after delivery (the national program recommends a minimum of 3 visits: the first, between 24 and 48 hours of delivery, the second between 7 and 14 days after delivery, and the third at 6 weeks after delivery), and infant immunization visits at 6, 10, and 14 weeks of age at the clinic (the Expanded Program on Immunization recommends bacille Calmette-Guérin (BCG)/polio/hepatitis B at birth and diphtheria, pertussis, and tetanus (DPT)/polio/hepatitis B/pneumonia at 6, 10, and 14 weeks). Participants completed a baseline interview upon recruitment and an endline interview at 14 weeks postpartum when they visited the center for completing the last of the primary vaccinations for infants. Monthly data were collected when clients visited the clinic for collecting monthly ART medications. Standardized data collection tools, staff training, and regular supervision ensured that study activities were uniform across sites.

### Description of the Intervention

#### Standard Care

All newly diagnosed pregnant women living with HIV (ART naïve) and those who became pregnant while on antiretroviral therapy (ART experienced) received routine HIV counseling from ART clinic-based counselors. The counseling included information on the risk of HIV transmission to the infant, the role of ART in PMTCT, the importance of adherence to treatment, disclosure and partner testing, institutional delivery, and infant HIV testing at 6 weeks postpartum. All participants received standard ANC services, which included blood pressure and weight measurements, hemoglobin, syphilis and urine testing, tetanus toxoid immunization, and iron and folic acid supplements. All participants also received standard PNC services, which included mother's check-up, HIV PCR testing for infants at 6 weeks postpartum, and routine immunization services. All centers had peer community health workers associated with the clinic to support clients and trace those who defaulted or missed visits.

#### Cell Phone Counseling Intervention

**FIGURE 1:**
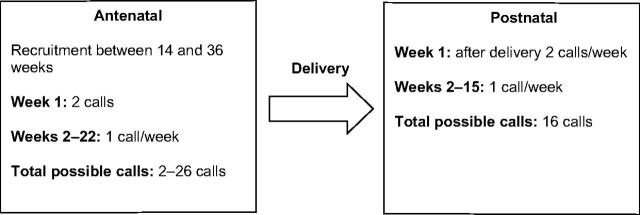
Cell Phone Counseling Protocol

In addition to standard care, participants in the intervention arm received one-on-one individualized counseling, delivered via cell phone by 5 trained counselors based at a central study office. The counseling was drawn from the Self-Regulation Theory, which is a system of conscious personal management that involves the process of guiding one's thoughts, behaviors, and feelings to empower patients to recognize their problems and find solutions.^21^ The sessions were structured to consist of 2 phone calls during the first week of starting PMTCT services, followed by 1 call/week until the participant delivered (maximum of 26 calls), followed by 2 calls during the first week after delivery and 1 call/week for 14 weeks thereafter (maximum of 16 calls) (Figure 1). The number of calls during the antenatal period varied between participants depending on when they presented for ANC services (between 14 and 36 weeks of gestation).

The cell phone counseling drew on the Self-Regulation Theory.

**Training of Counselors:** The counselors were trained HIV counselors who had at least 3 years of experience in HIV counseling at various testing centers and had completed at least high school education. The counselors received a 10-day training on the intervention that included theoretical background of the intervention, training on counseling techniques, role play with colleagues and trainers, and practice sessions with volunteers with HIV infection. During the first month, counselors were required to debrief with the program coordinator after each call and receive feedback. Thereafter, the counselors continued with a weekly group discussion on problem cases.

**Intervention** **Sessions:** After completing recruitment procedures, the research staff put the participants in touch with a study counselor via phone; the participants never met their counselor face-to-face during the entire study period. Participants were required to use their own phones, including a phone shared with a family member or friend. Counselors and patients decided mutually convenient times for the calls.

The first session focused on illness representation and problem identification. This was followed by the development and execution of a response plan and evaluation of coping strategies over follow-up sessions. Perceptions or contextual situations that could pose an impediment to ARV adherence or retention in care were identified and participants were encouraged to think about their experiences, interaction with others, sources of information, and cognitive and emotional processes that contributed to their perceptions. Participants were then encouraged to discuss strategies on how to manage their perceptions. Through this process, the counselors introduced replacement perceptions and alternate behaviors. The counselors helped participants address their areas of concern by providing targeted action plans, setting realistic goals, and assessing progress during the next follow-up call.

During the early antenatal period (14–32 weeks), counselors focused on the importance of adherence to treatment for their own health and to ensure their baby was born HIV-free. Partner disclosure, partner testing, stigma issues within the family/community, distance to ART centers, and travel constraints were assessed and participants counseled. The focus shifted to emphasize retention in care, institutional delivery, and the need for initiating nevirapine for the baby in the late antenatal period (32–40 weeks) while continuing to emphasize adherence. During the postnatal period (0–15 weeks postpartum), the counselors discussed nevirapine for the infant, infant feeding (exclusive breastfeeding), PCR testing of the infant at 6 weeks, completion of the primary immunization schedule of vaccines, and family planning for the mother while continuing to emphasize the need to continue ART and remain adherent. The Box details the topics covered during the calls.

BOX **Content Focus of Counseling Calls**

**Initial antenatal period** (14–32 weeks gestation)

- Personalized problem identification (first session)
- Importance of ART for PMTCT and infant outcomes
- Adherence to treatment, including monthly collection of medications and timely and regular intake of medication
- Discuss the importance of retention in care, including visit attendance
- Discuss partner involvement, HIV status, disclosure, and testing

**Late antenatal period** (32–40 weeks gestation)

- Emphasize retention in care
- Emphasize institutional delivery
- Introduce infant feeding, including exclusive breastfeeding
- Discuss nevirapine initiation for baby

**Postnatal period** (0–14 weeks postpartum)

- Confirm initiation and continuation of nevirapine for baby
- Infant feeding, including exclusive breastfeeding
- Infant HIV PCR testing at 6 weeks
- Completion of immunization, including BCG and polio at birth, DPT and polio at 6, 10, and 14 weeks
- Emphasize retention in care
- Introduce discussion on confirmatory HIV testing at 18 months
- Discuss family planning needs of the mother

Participants could make additional need-based calls to the counselor during working hours on weekdays to address concerns or queries. The calls enabled participants to have frequent, personalized, one-on-one contact with a health care professional without visiting the health facility. Data were collected on the frequency and duration of calls made, number of attempts made to reach the client, and reasons for unsuccessful calls. All study participants received a baby gift pack containing soap, baby oil, and disposable napkins when they came for their PNC visit at 6 weeks.

### Data Collection and Study Variables

Data were collected using structured questionnaires administered by research assistants in Swahili or Luo. Variables were categorized as follows: **education** as never attended school, received primary education, or attended secondary or university education; **marital status** as never married, married or cohabiting, or divorced/separated/widowed; **living arrangements** as lives alone, lives with partner/husband and children, or lives with others. **Pregnancy duration** at recruitment was categorized as 14–28 weeks or 29–36 weeks; **time since HIV-positive status** as 1 year or less, 2–4 years, or 5 years or more; and **partner/spouse's HIV status** as positive, negative, or unknown. Participants were considered **ART naïve** if they were diagnosed positive but had never received ART and experienced if they became pregnant while on ART; **PMTCT treatment regimens** were categorized as Option A or only AZT (zidovudine) for the mother, or Option B or combination of 3 ARVs for the mother; infants received nevirapine under Option A and nevirapine or AZT under Option B.^23^ **Depression** was assessed at baseline and endline using the Center for Epidemiologic Studies Depression (CES-D) scale, a 20-item validated scale.^24^ The scale has a possible range of scores of zero to 60 with higher scores indicating the presence of more symptomology. Depression was categorized as no depression if scores were <16 and depression if scores were ≥16. **Perceived stigma** was assessed at baseline and endline using a 16-item scale (Cronbach's alpha of adapted scale: 0.81), derived from Berger's HIV stigma scale^25^ that has been used in other studies in Kenya.^26^ The scale covered 4 domains: disclosure concerns, negative self-image, concerns about public attitudes, and personalized or experienced stigma. Total scores (range: 16–64) were categorized as low (16–40), moderate (41–52), or high (53–64) stigma. **General health perception** was assessed using the Health-Related Quality of Life tool used by AIDS Clinical Trials Group studies.^27^ The tool examines perceptions about general health; resistance to illnesses and health outlook; physical, social, role, and cognitive functioning; and pain. Item scores in each scale are summed to compute raw scale scores that are then transformed to a 0 to 100 scale. Higher scores are indicative of better health functioning. Scores were categorized as above average (61–100) or average or below (≤60). **Adherence** was assessed using the **Medication Possession Ratio (MPR**) derived from pharmacy refill information, collected from pharmacy registers, and recorded as a percentage.

MPR=Number of days participants had supply of medications/Number of days in the study.

For analysis, MPR was dichotomized as ≥90% or <90%.

**Retention in care** was assessed at 3 time points: at delivery, 6 weeks postpartum, and 14 weeks postpartum. Participants who delivered at the health facility where they received PMTCT services, or at another health facility, or for whom there was information of a home delivery and pregnancy outcome were considered retained at delivery. Participants who completed their 6-week PNC visit or had their baby tested for HIV (PCR test) or had the baby immunized at 6 weeks were considered retained at 6 weeks postpartum. Participants who had their baby immunized at 14 weeks were considered retained at 14 weeks postpartum. Participants with stillbirths and infant deaths prior to time points 6 weeks and 14 weeks postpartum were excluded from the analysis.

Retention in care was assessed at 3 time points: at delivery, 6 weeks postpartum, and 14 weeks postpartum.

Data were collected on HIV testing of the infant. In Kenya, the national program requires infants born to mothers living with HIV to undergo HIV PCR testing at 6 weeks after birth. We collected information on HIV PCR testing undertaken any time between 6 and 14 weeks postpartum from the child health register. Data on attendance at ANC and PNC services, including infant immunization, were collected from the maternal and child health register. Monthly ANC visits coincided with the ARV pick-up from the pharmacy; the number of ANC visits during the study period varied depending on when the participant registered for ANC.

**Counseling call details**, such as number of calls made and duration of each session, were recorded by the counselors and verified from itemized monthly statements.

### Statistical Analysis

Data were entered using the Census and Survey Processing (CSPro) software program (U.S. Census Bureau and ICF Macro) and analyzed using Intercooled Stata 10.0 (Stata Corporation, College Station, TX). Patients were analyzed within the group to which they were originally assigned. Unpaired Student's *t* test and Mann-Whitney U tests compared continuous variables with normal and non-normal distributions, respectively, and Pearson's chi-square test was used to compare categorical variables. We compared sociodemographic characteristics, HIV testing and disclosure, pregnancy history, and ART use between intervention and control participants to assess the effectiveness of randomization.

We had 2 primary outcomes for this study: retention in care until 14 weeks postpartum and uptake of HIV PCR testing in the infant (EID). For the first outcome, we compared participant retention at 3 discrete time points in the study period: at delivery, 6 weeks postpartum, and 14 weeks postpartum (the endpoint coinciding with the end of the first set of primary immunization for infants). To evaluate retention at 3 time points while taking into account factors associated with retention and time to lost to follow-up (LTFU), we used a complementary log-log regression model, which is an alternative extension of the proportional hazard model for discrete time survival analysis. We calculated the complementary log-log of the hazard function at the 3 defined time points using the following model:

$$\log\left[-\log\left\{\left[1-\lambda \right]\left(t_{j}|x_{i}\right)\right\}\right]=\alpha_{j}+\beta_{j}x_{i}$$

where:

- x_i_ is the vector consisting of sociodemographic, HIV–related, and study group variables for individual i
- β_j_ is the covariate matrix
- λ(t_j_|x_i_) is the hazard function for individual x_i_ at time point t_j_
- α_j_=log{−log(1− λ(t_j_|x_0_)} is the complementary log-log of the baseline hazard

From the above model, we calculated the hazard ratio of being LTFU for an individual ‘i’ compared to the reference category at time point ‘j’ using following equation:

$$\frac{\lambda \left(t_{j}|x_{i}\right)}{\lambda \left(t_{j}|x_{0}\right)}=1-e^{-e^{\alpha_{j}+\beta_{j}x_{i}}}$$

For each predictor variable, the baseline models were controlled for the time variable (the 3 time points), age, education, and marital status. We also examined the interaction effect of the predictor variable with the time variable. In the results, we display the interaction term only when found significant (*P*<.05). The final model is a multivariate model controlling for age, education, and marital status. This model includes only the variables that were statistically significant in the baseline model.

Pearson's chi-square test was used to compare the uptake of EID between the intervention and control arms. The incidence rate for HIV transmission among infants was calculated over the time period from birth to date of HIV PCR test by dividing the number of new infections by the total weeks of exposure. We used binary logistic regression to identify the predictors of HIV infection among infants.

We compared the uptake of ANC and PNC services among participants in the 2 groups using Pearson's chi-square test. We also provide relative risk ratios (RRRs) for not attending at least 50% of the required visits (the number varied depending on when the participant registered for ANC), not taking the complete ANC package, not completing 3 PNC visits, not attending the 6-week PNC visit, and not delivering at a health facility in the intervention arm compared with the control arm.

### Ethical Considerations

The study was approved by the Kenyatta National Hospital, University of Nairobi Ethics and Research Committee and the Institutional Review Board of the Population Council. All participants provided written informed consent.

## RESULTS

### Baseline Characteristics of Subjects

**FIGURE 2:**
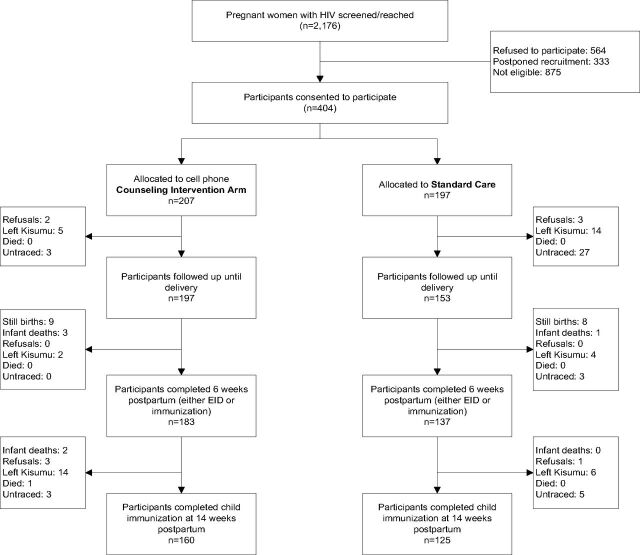
Flow Diagram of Participant Recruitment and Follow-Up in the Healthy Mother Healthy Baby Project in Kisumu, Kenya (2013–2016) Abbreviation: EID, early infant diagnosis.

A total of 2,176 pregnant women living with HIV were screened at 14 PMTCT-ART centers in and around Kisumu County. Among those initially screened, 564 women refused to participate, 333 agreed to participate at a later date but never returned to complete recruitment procedures, and 875 were ineligible per the eligibility criteria, including 355 women who did not own or have access to a cell phone. In total, we recruited 404 pregnant women living with HIV; 207 were randomly assigned to the intervention arm and 197 to standard care (Figure 2).

There were no significant differences in sociodemographic and pregnancy-related characteristics between the intervention and control groups at baseline (Table 1), indicating effective randomization. In both groups combined, 57% of women were ART naïve, 21% had received ARVs for a previous pregnancy, and 22% were on ART for treatment of their infection (CD4 cell count <500 cells/ml). Two-thirds of the women had been diagnosed with HIV infection in the past 1 year and 36% had a spouse with HIV while nearly half (47%) did not know the HIV status of their spouse/partner.

**TABLE 1.** Baseline Characteristics of Participants Recruited in Kisumu, Kenya (2014)

	Intervention (n=207)	Control (n=197)	Total (N=404)	*P* Value^a^
**Age, years, median (IQR)**	24 (22, 28)	25 (22, 29)	25 (22, 29)	
**Educational level, n/N (%)**				
No education or adult literacy	4/207 (1.9)	3/197 (1.5)	7/404 (1.7)	.72
Primary/preschool	133/207 (64.3)	134/197 (68.0)	267/404 (66.1)	
Secondary or higher	70/207 (33.8)	60/197 (30.5)	130/404 (32.2)	
**Current marital status, n/N (%)**				
Married/cohabiting	169/207 (81.6)	164/197 (83.3)	333/404 (82.4)	.31
Never married	25/207 (12.1)	16/197 (8.1)	41/404 (10.2)	
Divorced/separated/widowed	13/207 (6.3)	17/197 (8.6)	30/404 (7.4)	
**Living arrangements, n/N (%)**				
Lives by herself	12/207 (5.8)	14/197 (7.1)	26/404 (6.4)	.25
Lives with partner/spouse/children	155/207 (74.9)	157/197 (79.7)	312/404 (77.2)	
Lives with other relatives/friends	40/207 (19.3)	26/197 (13.2)	66/404 (16.3)	
**Employment status, n/N (%)**				
Employed	25/207 (12.1)	39/197 (19.8)	64/404 (15.8)	.03
Not employed	182/207 (87.9)	158/197 (80.2)	340/404 (84.2)	
**Home district, n/N (%)**				
Kisumu	60/207 (29.0)	67/197 (34.0)	127/404 (31.4)	.28
Others	147/207 (71.0)	130/197 (66.0)	277/404 (68.6)	
**Pregnancy duration at recruitment, n/N (%)**				
14-28 weeks	161/207 (77.8)	155/197 (78.7)	316/404 (78.2)	.83
29-36 weeks	46/207 (22.2)	42/197 (21.3)	88/404 (21.8)	
**Total pregnancies, including current, median (IQR)**	3 (2, 4)	3 (2, 4)	3 (2, 4)	.38
**Total number of children**				
Living, mean (SD)	1.0 (1.5)	1.7 (1.0)	1.8 (1.3)	.045
Dead, mean (SD)	1.2 (1.6)	1.4 (1.3)	1.3 (1.5)	.34
**Duration of HIV+ status, n/N (%)**				
1 year or less	136/207 (65.7)	132/197 (67.0)	268/404 (66.3)	.63
2–4 years	49/207 (23.7)	40/197 (20.3)	89/404 (22.0)	
5 or more years	22/207 (10.6)	25/197 (12.7)	47/404 (11.6)	
Disclosed status to partner/spouse, n/N (%)	120/207 (58.0)	116/197 (58.9)	236/404 (58.4)	
**Knows the HIV status of spouse/partner, n/N (%)**				
Yes	110/207 (53.1)	105/196 (53.6)	215/403 (53.4)	.93
Don't know	97/207 (46.9)	91/196 (46.4)	188/403 (46.7)	
**HIV status of spouse/partner, n/N (%)**				
Positive	77/110 (70.0)	76/105 (72.4)	153/215 (71.2)	.70
Negative	33/110 (30.0)	29/105 (27.6)	62/215 (28.8)	
**ART use, n/N (%)**				
Naïve	118/207 (57.0)	113/197 (57.4)	231/404 (57.2)	.94
Experienced	89/207 (43.0)	84/197 (42.6)	173/404 (42.8)	
**Type of ARV treatment assigned at PMTCT, n/N (%)**				
Option A (only AZT)	84/207 (40.6)	84/197 (42.6)	168/404 (41.6)	.88
Option B (3 ARVs)	123/207 (59.4)	113/197 (57.4)	236/404 (58.4)	
**CD4 cell counts at baseline,^b^ n/N (%)**				
<350 cells/ml	50/162 (30.8)	53/156 (34.0)	103/318 (32.4)	.64
351–500 cells/ml	37/162 (22.8)	39/156 (25.0)	76/318 (23.9)	
>500 cells/ml	75/162 (46.3)	64/156 (41.0)	139/318 (43.7)	
**Viral load at baseline,^c^ n/N (%)**				.38
<1000 copies/ml	70/90 (77.8)	60/72 (83.3)	130/162 (80.3)	
>1001 copies/ml	20/90 (22.2)	12/72 (16.7)	32/162 (19.8)	
**Stigma score (16–64), n/N (%)**				
Low (16–40)	146/207 (70.5)	135/197 (68.5)	281/404 (69.6)	.32
Moderate (41–52)	59/207 (28.5)	62/197 (31.5)	121/404 (30.0)	
High (53–64)	2/207 (1.0)	0/197 (0.0)	2/404 (0.5)	
**Depression score (10–60), n/N (%)**				
No depression (<16)	136/207 (65.7)	107/197 (54.3)	243/404 (60.2)	.02
Sign of depression (≥16)	71/207 (34.3)	90/197 (45.7)	161/404 (39.9)	
**General health perception (0–100), n/N (%)**				
Good/excellent (>60)	99/207 (47.8)	76/197 (38.6)	175/404 (43.3)	.06
Average/poor/very poor (≤60)	108/207 (52.2)	121/197 (61.4)	229/404 (56.7)	

Abbreviations: ART, antiretroviral therapy; ARV, antiretroviral; AZT, zidovudine; CD4, cluster of differentiation 4; IQR, interquartile range; PMTCT, prevention of mother-to-child transmission; SD, standard deviation.

a *P* value is from chi-square test for discrete variables; Mann-Whitney U test for median and *t* test in case of means for continuous variables.

b CD4 cell counts were obtained from clinic records; there are missing values due to missing records.

c Viral load tests were initiated late in the course of the study, thus results are available for only some participants.

### Exposure to Intervention

Cell phones were not provided to the study participants; 68% of the intervention group participants used their own cell phone, 19% reported using their spouse's cell phone, 8% used another family member's phone, and 5% relied on friends. Reaching participants via phone calls was challenging and counselors had to make multiple calls to reach clients. It took an average of 4.8 call attempts (standard deviation [SD]=7.9) to make a successful call to participants. Overall, participants attended an average of 63% (SD=24.6) of the required number of counseling sessions via phone calls during the study period, with a counseling session lasting an average of 9.2 minutes (SD=7.9). Just over one-third (37%) of participants attended more than 75% of the required number of sessions/calls (Table 2). The average duration of the calls was higher among participants who attended fewer calls. Further, the average duration of counseling sessions was longer for participants with symptoms suggestive of depression (CES-D scale score≥16) compared with those without depression (10.8 minutes vs. 8.4 minutes, respectively; *P*<.001; data not shown).

**TABLE 2.** Intervention Exposure and Retention Among Intervention Participants (n=207)

Percentage of Required Counseling Sessions Attended	Distribution of Participants Who Attended Calls (%)	Duration of Calls (minutes)^a^ Mean (SD)	Participants Retained Until 14 Weeks Postpartum^b^ (%)
≤25%	10.7	18.7 (19.5)	55.6
26%–50%	17.0	10.5 (4.8)	63.3
51%–75%	35.0	7.0 (2.7)	92.7
>75	37.4	7.9 (4.2)	89.3

Abbreviations: ANOVA, analysis of variance; SD, standard deviation.

a One way ANOVA. Differences between categories were significant at *P*<.001.

b Chi-square test. Differences between categories were significant at *P*<.001.

Counselors made an average of 4.8 attempts before placing a successful call to participants.

### Effects of Cell Phone Counseling on Participant Retention

**FIGURE 3:**
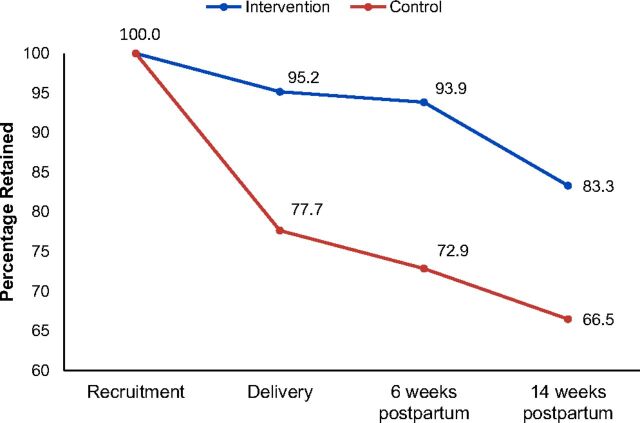
Participant Retention in Care at Delivery and 6 Weeks and 14 Weeks Postpartum, Kisumu, Kenya Followed until delivery: intervention, 197/207; control, 153/197. Followed until 6 weeks postpartum: intervention, 183/195; control, 137/188. Denominators exclude 17 participants total with still births (intervention, 9; control, 8) and 4 participants with infant deaths (intervention, 3; control, 1). Followed until 14 weeks postpartum: intervention, 160/192; control, 125/188. Denominator for the intervention arm excludes 2 participants with infant deaths after 6 weeks postpartum.

Participant retention in care was significantly higher in the intervention arm than the control arm at all 3 time points—at delivery: 95.2% vs. 77.7%; at 6 weeks: 93.9% vs. 72.9%; and at 14 weeks: 83.3% vs. 66.5% (Figure 3). All differences were significant at the *P*<.001 level. The highest dropout (44 participants; 22%) was observed before delivery among participants receiving standard care (Figure 2). This was followed by about 10.4% (20 participants) in the intervention arm who dropped out between 6 and 14 weeks postpartum (2 women with infant deaths and 1 woman who died after 6 weeks postpartum were excluded). In both arms, the dropout rate was lowest during the period between delivery and 6 weeks postpartum.

Retention in care was significantly higher in the intervention arm than the control arm at all 3 time points.

In the intervention arm, we observed a trend toward a linear relationship between retention and attendance at counseling calls, with higher retention among participants who attended a higher proportion of counseling calls (Table 2). The highest retention (92.7%) was observed among participants who attended between 51% and 75% of the calls. Among participants who were LTFU, the majority (70%) owned the cell phones they used. The use of shared phones did not have any effect on LTFU rates.

### Predictors of Loss to Follow-Up

In the baseline complementary log-log models, being employed (hazards ratio [HR]=1.69; 95% confidence interval [CI]=1.07, 2.33) and higher depression scores (HR=1.02; 95% CI=1.00, 1.03) were significant predictors of higher hazards of being LTFU (Table 3). On the other hand, knowing one's HIV status for 5 or more years (HR=0.62; 95% CI=0.39, 0.97), having disclosed their HIV status to their partner (HR=0.68; 95% CI=0.44, 1.00), knowing that their partner has HIV compared with not knowing their partner's HIV status (HR=0.62; 95% CI=0.38, 0.96), having better general health perception (HR=0.98; 95% CI=0.97, 0.99), and being in the intervention group (HR=0.22; 95% CI=0.11, 0.46) were significantly associated with lower hazards of being LTFU. Compared with the antenatal period, the lowest risk of being LTFU was in the 6-week postpartum period (HR=0.21; 95% CI=0.10, 0.43), followed by the period between 6 and 14 weeks postpartum (HR=0.76; 95% CI=0.49, 1.13; not statistically significant). Overall, participants in the intervention group had a lower hazard of being LTFU (HR=0.22; 95% CI=0.11, 0.46) and LTFU was lower during the 2 postpartum periods (delivery to 6 weeks: HR=0.21; 6 to 14 weeks: HR=0.76). However, the interaction term with time was significant for the study group; thus, between the 2 postpartum time periods, participants in the intervention group had a higher hazard of dropping out in the period between 6 and 14 weeks postpartum.

**TABLE 3.** Predictors of Loss to Follow-Up at Delivery and 6 and 14 Weeks Postpartum (Complementary Log-Log Regression Model)

	Baseline Model (N=404)		Final Model (N=404)	
	HR (95% CI)	*P* Value	HR (95% CI)	*P* Value
**Time point**				
Delivery	1		1	
6 weeks postpartum	0.21 (0.10, 0.43)	<.001	0.27 (0.10, 0.70)	<.001
14 weeks postpartum	0.76 (0.49, 1.13)	.18	0.47 (0.22, 0.89)	.003
**Living arrangements**				
Lives with partner/spouse/children	1			
Lives by herself or with other relatives/friends	0.74 (0.29, 1.65)	.50		
**Employment status**				
Not employed	1		1	
Employed	1.69 (1.07, 2.33)	.03	1.2 (0.81, 1.13)	.28
**Home district**				
Kisumu	1			
Other	1.22 (0.80, 1.73)	.35		
**Pregnancy duration at recruitment**				
14–28 weeks	1			
29–36 weeks	1.08 (0.69, 1.58)	.73		
Total pregnancies, including current	1.00 (0.90, 1.10)	.95		
**Has had HIV-affected child (living/died)**				
No	1			
Yes	0.89 (0.47, 1.56)	.72		
**Duration of knowing own HIV status**				
<5 years	1		1	
≥5 years	0.62 (0.39, 0.97)	.04	0.83 (0.49, 1.06)	.33
**Disclosure of HIV status to spouse/partner**				
Not disclosed	1		1	
Disclosed	0.68 (0.44, 1.00)	0.05	0.93 (0.58, 1.09)	.68
**Awareness of partner's HIV status**				
Does not know	1		1	
Knows, partner positive	0.62 (0.38, 0.96)	.03	0.81 (0.47, 1.06)	.27
Knows, partner negative	0.81 (0.45, 1.34)	.46	0.99 (0.54, 1.12)	.97
Stigma score (16–64) at baseline	1.01 (0.98, 1.04)	.57		
Depression score (10–60) at baseline	1.02 (1.00, 1.03)	.046	1.00 (0.98, 1.00)	.55
General health perception (0–100) at baseline	0.98 (0.97, 0.99)	<.001	0.99 (0.98, 1.00)	.01
**Experience of physical/sexual violence from partner at baseline**				
Never	1			
At least once	1.33 (0.90, 1.81)	.15		
**Baseline viral load**				
<1000	1			
≥1000	1.02 (0.40, 1.86)	.96		
**CD4 count at enrollment**				
≤350	1			
351–500	0.70 (0.34, 1.33)	.30		
>500	0.81 (0.45, 1.36)	.47		
**ART experience**				
Naïve	1			
Experienced	0.80 (0.52, 1.17)	.26		
**Study group assignment**				
Control	1		1	
Intervention	0.22 (0.11, 0.46)	<.001	0.29 (0.12, 0.69)	<.001
**Interaction between time point and study group**				
Delivery	1		1	
6 weeks postpartum * Intervention	1.10 (0.21, 2.33)	.90	1.05 (0.22, 1.14)	.93
14 weeks postpartum * Intervention	3.35 (2.19, 2.41)	<.001	1.86 (2.01, 1.14)	<.001

Abbreviations: ART, antiretroviral therapy; CD4, cluster of differentiation 4; CI, confidence interval; HR, hazard ratio.

Note: Both baseline and final models are controlled for age, education, and marital status. All baseline models were tested for interaction effect with the time variable. The time interaction effect is included only if it is significant (*P*<.05). Final model comprised of variables that were significant in the baseline models.

The final regression model shows that the conditional hazards of being LTFU during the postpartum period were significantly lower compared with the antenatal period: for 6 weeks postpartum (HR=0.27; 95% CI=0.10, 0.70) and for the 6–14-week period (HR=0.47; 95% CI=0.22, 0.89). Many of the variables that were significantly associated with LTFU in the baseline model lost their significance in the final model, except for general health perception and the study group. Participants with better health perceptions had a lower hazard of being LTFU (HR=0.99; 95% CI=0.98, 1.00). Being in the intervention group had a significantly lower hazard of being LTFU overall (HR=0.29; 95% CI=0.12, 0.69). However, a significant interaction term shows that the comparative hazard of being LTFU for intervention versus the control group was higher between 6 and 14 weeks postpartum (as reported earlier, the second largest number of participants LTFU were in the intervention group in the 6–14-week period).

Positive health perceptions and being in the intervention arm were significantly associated with lower loss to follow-up.

### Effect of Cell Phone Counseling on EID Uptake

Uptake of infant HIV testing was significantly higher among participants in the intervention arm compared with those in standard care (92.8% vs. 68.1%, respectively; *P*<.001), followed until 14 weeks postpartum (Table 4). Stillbirths (n=9 in intervention; n=8 in control) and infant deaths (n=3 in intervention; n=1 in control) prior to 6 weeks were excluded from this analysis. PCR testing was conducted at a median of 44 days postpartum (interquartile range [IQR]=42, 49). The time of HIV testing of infants did not differ between the 2 groups. PCR test results were available for 308 infants. Of these, 9 infants (2.9%) tested positive, 7 infants in the intervention group and 2 infants in the control group (HIV incidence rate=0.39 per 100 infant-weeks; 95% CI=0.20, 0.75) (Table 5).

**TABLE 4.** Uptake of EID, HIV Status, HIV Incidence, and Predictors of HIV Infection Among Infants

	Intervention n/N (%)	Control n/N (%)	*P* Value
**Uptake of EID**			
EID conducted^a^	181/195 (92.8)	128/188 (68.1)	<.001
EID conducted within 8 weeks^b^	145/181 (80.1)	104/128 (81.2)	.25
**HIV status of infant^c^**			
HIV negative	174/181 (96.1)	125/127 (98.4)	
HIV positive	7/181 (3.9)	2/127 (1.6)	.80

Abbreviations: EID, early infant diagnosis; PCR, polymerase chain reaction.

a Assessed among those who were followed until 14 weeks postpartum, excluding 17 women with stillbirths and 4 women whose infant died before reaching 6 weeks postpartum who did not need to return for EID.

b Assessed among infants who were tested for HIV.

c Assessed among those for whom PCR test results were available.

**TABLE 5.** HIV Incidence Rate by Study Group

	Total Infants	Total Incidents	Total Infant Weeks	Incidence Rate/100 Infant-Weeks (95% CI)
Control	181	2	962	0.21 (0.05, 0.83)
Intervention	127	7	1333	0.53 (0.25, 1.10)
Total	308	9	2295	0.39 (0.20, 0.75)

Abbreviation: CI, confidence interval.

On binary logistic regression analysis, lower MPR, indicating poor adherence, was the main predictor of HIV infection among infants (Table 6). Overall, 20.8%, 28.4%, and 17.5% of the participants had MPR less than 90% at delivery, 6 weeks postpartum, and 14 weeks postpartum, respectively; there was no difference between the intervention and control groups (data not shown). Among the 9 infants who tested HIV positive, for 6 of the infants, the mother had MPR<90% at delivery and at 6 weeks postpartum.

**TABLE 6.** Determinants of HIV Infection Among Exposed Infants (Binary Logistic Regression Model)

	Adjusted OR (95% CI)	*P* Value
**ART experience**		
Naïve	Ref	
Experienced	1.90 (0.39, 9.15)	.42
**Pregnancy duration at recruitment**		
14–28 weeks	Ref	
29–36 weeks	0.57 (0.06, 5.22)	.62
**Place of delivery**		
Home	Ref	
Institutional	0.61 (0.05, 7.64)	.70
**Infant feeding**		
Mixed or complementary feeding	Ref	
Exclusive breastfeeding	0.26 (0.02, 3.00)	.28
**Medication Possession Ratio**		
≤90% or less	Ref	
>90%	0.20 (0.04, 0.99)	.05
**Study group assignment**		
Control	Ref	
Intervention	6.43 (0.70, 59.44)	.10

Abbreviations: ART, antiretroviral therapy; CI, confidence interval; OR, odds ratio.

Note: Regression model is adjusted for mother's age and education.

### Uptake of Maternal Health Services

The ANC attendance rates were higher among participants in the intervention arm than the control arm; 54.6% of the participants in the intervention arm completed more than 75% of the required number of visits compared with 41.8% in the control arm (*P*=.03) (Table 7). The required number of visits varied for participants depending on when in their pregnancy they registered for ANC services. The relative risk ratio of not completing at least 50% of the visits was lower in the intervention arm (RRR=0.88; 95% CI=0.80, 0.98) compared with the control group.

**TABLE 7.** Uptake of Maternal and Child Health Services and Infant HIV PCR testing

	Intervention n/N (%)	Control n/N (%)	*P* Value^a^	Unadjusted RRR (95% CI)
**Attended^b^:**				
50% or less of required ANC visits	60/187 (32.1)	81/182 (44.5)		0.88 (0.80, 0.98)
51%–75% of required ANC visits	25/187 (13.4)	25/182 (13.7)	.03	
76%–100% of required ANC visits	102/187 (54.6)	76/182 (41.8)		
**Received complete ANC package^c^**	192/207 (92.8)	181/196 (92.4)	.88	1.00 (0.95, 1.05)
**Attended 3 PNC visits per national protocol**	42/207 (20.3)	25/197 (12.7)	.04	0.93 (0.86, 1.00)
**Attended 6-week PNC visit**	170/207 (82.1)	139/197 (70.6)	.006	0.89 (0.82, 0.97)
**Delivered at a health facility^d^**	188/197 (95.4)	143/153(93.5)	.42	0.98 (0.93, 1.03)
**Infants with full primary immunization^e^**	156/160 (97.5)	121/125 (96.8)	.72	1.01 (0.97, 1.05)

Abbreviations: ANC, antenatal care; BCG, bacille Calmette-Guérin; CI, confidence interval; DPT, diphtheria, pertussis, and tetanus; PNC, postnatal care; RRR, relative risk ratio.

a Chi-square test.

b Assessed among those who required at least 1 ANC visit from the time of recruitment until delivery.

c ANC package was considered complete if the following were done: hemoglobin, venereal disease research labs, blood group, and at least 1 urine test.

d Assessed among those who were followed until delivery.

e Full primary immunization was considered if the infant received BCG + polio at birth, and DPT + polio at 6, 10, and 14 weeks of age.

ANC attendance was higher in the intervention arm than the control arm.

For complete PNC coverage, the national program requires women who deliver to be seen within 24 to 48 hours after delivery followed by visits between 7 and 14 days postpartum and at 6 weeks postpartum. PNC attendance rates (3 PNC visits) were higher among participants in the intervention arm compared with the control (20% vs. 13%, respectively; RRR of non-completion of 3 PNC visits, intervention vs. control=0.93; 95% CI=0.86, 1.00) (Table 7). Attendance at the 6-week PNC visit that coincides with HIV testing of HIV-exposed infants was also higher in the intervention arm than the control (82% vs. 71%, respectively; RRR of non-attendance, intervention vs. control=0.89; 95% CI=0.82, 0.97).

## DISCUSSION

In this randomized study, we demonstrated that one-on-one individually tailored, theory-based counseling delivered via cell phone was highly effective in retaining mothers with HIV infection in care at delivery and at 6 and 14 weeks postpartum compared with standard care. About one-quarter of participants in the control group were LTFU in the period between recruitment and delivery. The number of dropouts reduced significantly after delivery indicating that **the risk of LTFU is greatest prior to delivery**. Most studies have examined retention in the postpartum period; non-retention in the antenatal period requires special attention. Retention also declined between 6 and 14 weeks postpartum (20 women moved out of Kisumu, 14 in the intervention arm and 6 in the control arm; 8 women were untraced, 3 in the intervention arm and 5 in the control). The women appeared to have waited for the HIV test results before moving out of Kisumu, suggesting that EID is a critical follow-up point in PMTCT programs.

The one-on-one individually tailored cell phone counseling intervention was highly effective in retaining mothers with HIV in care at delivery and at 6 and 14 weeks postpartum.

The counseling intervention and positive health perceptions were independent predictors for participant retention at all 3 time points in the study. However, it is important for program managers to also focus on the other factors found to *negatively* influence retention in the baseline models controlled for age, education, marital status, and the intervention. These factors included the presence of depression and being employed, even though these factors were not significant in the multivariate model. A higher proportion of women in the control arm were employed and being employed was a predictor of loss to follow-up in the baseline model. It is possible this may have contributed to some extent to the higher LTFU rates in the control group. Being employed would make it difficult for women to attend clinic services or take calls. Program managers and health workers need to accommodate the time constraints and needs of employed women to improve service uptake. Women who had disclosed their status to their spouses and those who knew the HIV status of their spouse (especially those with a spouse who had HIV) were less likely to be lost to follow-up. Disclosure and knowledge of the HIV status of the spouse would have made it easier for women to receive counseling calls, talk freely, attend scheduled clinic visits, and get their infant tested for HIV infection on time. It is important for health workers to encourage and support disclosure and partner testing. We documented a trend toward a linear relationship in the effect of the intervention—**retention increased with higher exposure to counseling**—suggesting a benefit of the ongoing relationship between the women and their cell phone counselors. The counseling intervention was also highly effective in promoting the uptake of EID; we observed a significantly **higher uptake of EID among participants in the intervention arm** compared with the control arm.

With the widespread use of cell phones in Africa, other studies have evaluated the use of cell phone technology for health messaging to promote retention in care, but almost all studies have used SMS. This is the first study to provide focused and individualized counseling delivered via phone technology from one offsite central location. We report much higher retention rates at 6 weeks than a 2-arm randomized controlled study by Odeny et al. (2014) that used SMS to increase attendance at maternal postpartum clinic (intervention vs. control: 19.6% vs. 11.8%; relative risk=1.66; 95% CI=1.02, 2.70) in the Nyanza region of Kenya.^3^ A second, more recent randomized cluster study, conducted in Homa Bay, Nyanza, by Kassaye et al. (2016), used 2-way SMS between counselors and patients to improve retention, uptake of EID, and face-to-face communication.^16^ The authors report high retention at 6 weeks in both the intervention arm (87%; 244/280) and the control arm (84%; 227/270), and similarly improved communication between patients and counselors in both arms. Both studies report very high rates for infant testing (Odeny et al.: intervention vs. control, 92% vs. 85%; Kassaye et al.: intervention vs. control, 88% vs. 89%) and low HIV positivity among infants tested (Odeny et al.: 1.5%; Kassaye et al.: 0.9%). In contrast, we report significantly higher HIV testing in the intervention arm (94%) compared with standard care (68%), but also higher positivity rates with more HIV PCR-positive infants in the intervention arm than the control arm (2.9%; n=7 in the intervention and n=2 in the control). Differential follow-up rates in the 2 arms in our study may have contributed to these results—that is, a higher proportion of participants in the control arm were not retained and their infants were not tested. It is also possible that there may be programmatic differences in the 2 geographic areas, Homa Bay and Kisumu, where the studies have been conducted. Both are located in the same province on the shores of Lake Victoria, but Homa Bay is a high HIV prevalence area with a long-standing, mature HIV program with several programmatic interventions in place and a population familiar with HIV. Although there was no significant difference between the 2 groups of participants with regard to MPR, we found lower MPR to be an independent predictor of HIV infection among infants. Thus, while retention and uptake of EID improved with our intervention, adherence remained a concern, especially in about one-fifth of the participants. Our findings highlight the need to closely follow pregnant women living with HIV in PMTCT programs, especially during the antenatal period and after EID (around the 6-week postnatal visit) with an increased focus on collection of medication and adherence to treatment. HIV transmission continues to remain a serious concern.

This is the first study to provide focused and individualized counseling delivered via mobile phones.

The counseling intervention resulted in higher antenatal and postnatal attendance rates in the intervention group compared with the control group. Interestingly, our postnatal attendance rates at 6 weeks (intervention vs. control, 80.1% vs. 70.5%) were higher than those reported by the 2014 Kenya Demographic and Health Survey for the general population of women in Nyanza and Kenya (36.7% women in Nyanza and 43.0% in Kenya had no PNC visit at all).^28^ Similarly, institutional delivery rates were similar across the 2 groups (average 94.4%) but significantly higher than institutional delivery rates reported by the Demographic and Health Survey among the general population of women in Kisumu (69.5%) and Kenya (61.2%).^28^ It appears that mothers living with HIV who are enrolled in the PMTCT program are more likely to obtain maternal health care services than the general population.

Although cell phone coverage has increased dramatically across many countries in the continent and is reported to be around 80% in Kenya, many women (355 women) did not have access to cell phones and thus could not participate in the study and benefit from the intervention. Further, counselors found it challenging to reach participants and make calls, taking on average more than 4 attempts to make a successful call. Finding a suitable window of time for the client to be able to talk freely and having access to phones shared with a spouse or family member are substantial barriers. As the intervention was found to be very effective in retaining patients in care and promoting uptake of EID, programs implementing this intervention may consider the provision of cell phones to women who are at higher risk of loss to follow-up and do not have access to a phone. The intervention tested is resource intensive, which may be a concern for programs considering scale up. Programs could consider reducing the total number of calls by about 30% based on the data that shows that the highest retention was observed among participants who attended between 51% and 75% of the calls. Alternatively, programs may consider allocating the intervention only to participants at higher risk of loss to follow-up, such as those reporting depressive symptoms or those who may not have disclosed their HIV status to their partners/families. Rigorously monitored program data can then be used to assess impact at scale.

To make it easier to scale-up the intervention, programs could consider reducing the total number of calls or allocating the intervention only to participants at higher risk of loss to follow-up.

### Limitations

The study is not without limitations. We observed a very high rate of refusal during the screening process (564 refusals and 333 women who postponed recruitment and did not return), indicating a reluctance to be identified and contacted regularly, suggesting that stigma is still deeply entrenched in the community. Other studies have also reported persistent HIV-related stigma in African communities and the role of stigma in lower uptake of PMTCT services and EID in Kenya.^29^^–^^31^ Efforts must be made to reduce stigma in community. It is of note, however, that among study participants, stigma did not have an effect on retention or uptake of EID. As the intervention moves from a research setting to program delivery when scaled up, a high refusal rate has the potential to attenuate the population-level impact of the intervention. A detailed discussion of the program and its benefits (personalized and confidential access to counselors, information and support) by clinic staff may help to overcome stigma and increase participation, especially among women who are vulnerable and more likely to be lost to follow-up. Further, the lack of access to cell phones limited inclusion of a large number of women, which could have biased the sample. Therefore, the results should be interpreted within the given context. In our assessment of retention, we excluded women who had a stillbirth or infant death prior to 6 and 14 weeks postpartum, as these women would not need to attend child care services. This could have led to an underestimation of the infant HIV positivity rate as the stillbirths or infant deaths could have been due to in utero HIV infection. Lastly, we used MPR as a measure of adherence; it is, however, important to note that collection of medications does not confirm that participants consumed the medications. Future studies should include a more direct measure of adherence such as therapeutic drug monitoring or ante- and postnatal viral load monitoring.

## CONCLUSION

In conclusion, the one-on-one individually tailored, theory-based counseling delivered via cell phone was very effective in retaining mothers with HIV in care and in promoting the uptake of EID and antenatal and postnatal care services. Within the intervention, a greater emphasis is required on the collection of medications and adherence.

## Notes

**Peer Reviewed**

**First Published Online:** May 29, 2019

**Funding:** This study was made possible through support provided by the U.S. President's Emergency Plan for AIDS Relief and the U.S. Agency for International Development (USAID) via HIVCore, a Task Order funded by USAID under the Project SEARCH indefinite quantity contract (contract no. AID-OAA-TO-11-00060). The Task Order is led by the Population Council in partnership with the Elizabeth Glaser Pediatric AIDS Foundation, Palladium and the University of Washington. The content of this article is the responsibility of the authors and does not necessarily reflect the views of USAID or the U.S. government.
